# Robotic single-port (SP) vaginal hysterectomy: the first cases in Europe and surgical steps

**DOI:** 10.1007/s00464-026-12883-6

**Published:** 2026-05-12

**Authors:** Layanna Otrante, Louise Moniod, Clement Rousseau, Celine Chauleur

**Affiliations:** 1 Department of Gynecology and Obstetrics, Saint-Etienne University Hospital, Avenue Albert Raimond, 42270 Saint Priest en Jarez, France; 2 INSERM, U1059, 42023 Saint-Étienne, France

**Keywords:** vNOTES, Single port, Robotic vaginal surgery, Hysterectomy

## Abstract

**Background:**

Vaginal hysterectomy is the preferred approach when feasible, but technical limitations may restrict its use in complex cases. Robotic assistance combined with vaginal natural orifice transluminal endoscopic surgery (vNOTES) aims to overcome these limitations. The da Vinci® Single-Port (SP) system, designed for narrow spaces, may further optimize robotic-assisted vaginal surgery.

**Methods:**

We conducted a retrospective, single-center case series including all robotic-assisted vaginal hysterectomies performed using the da Vinci® SP system (RSP-vNOTES) between October 2024 and April 2026. Clinical, perioperative, and postoperative data were collected from medical records. Outcomes were analyzed using descriptive statistics.

**Results:**

Seventeen RSP-vNOTES hysterectomies were performed by two experienced surgeons. Median total operative time was 74 min (60.8, 96.8). The main surgical indication was uterine fibroids with large uterine volume; median uterine weight was 370 g (61, 800). Estimated blood loss was minimal, with no conversions or intraoperative complications. Sixteen patients (94.1%) underwent same-day discharge; one patient required one night hospitalization due to anesthetic contraindications related to comorbidities. Postoperative pain was low, with a median VAS score of 0 at 4 h (0–1). No postoperative complications were observed within 30 days.

**Conclusion:**

Robotic-assisted vaginal hysterectomy using the da Vinci® Single-Port system is feasible and safe, with favorable short-term outcomes, including in cases with large uterine volume. Direct intravaginal insertion of the SP access port provides effective robotic assistance in a confined anatomical space. Larger prospective studies are needed to confirm these findings and further define the role of single-port robotic technology in vaginal gynecologic surgery.

**Supplementary Information:**

The online version contains supplementary material available at 10.1007/s00464-026-12883-6.

Hysterectomy is one of the most frequently performed gynecological surgeries worldwide. When feasible, the vaginal route is considered the gold standard. Nevertheless, the laparoscopic approach remains preferred by many surgeons, providing better visualization and facilitates the learning process [1, 2].

Recently, the development of vaginal Natural Orifice Transluminal Endoscopic Surgery (vNOTES) represents a significant advancement in gynecological minimally invasive surgery by combining the benefits of both vaginal and laparoscopic approaches [3–7]. Several studies have demonstrated that vNOTES hysterectomy is associated with reduced postoperative pain, shorter hospital stay, faster recovery, and improved cosmetic outcomes compared with abdominal approaches [7–9]. However, technical limitations persist, particularly in case involving large uterine volumes.

The integration of robotic assistance into vNOTES (RvNOTES) has been proposed to overcome these limitations and broaden the indications for vaginal hysterectomy. Feasibility has been demonstrated using multi-arm robotic platforms such as the da Vinci® Xi system [10–14], but in our practice, instrument collisions limit the maneuverability in the vaginal space. Moreover, the multi-arm configuration of these platforms poses inherent ergonomic challenges when deployed through the transvaginal route. The da Vinci® Single-Port (SP) system, with its articulated camera and instruments designed to facilitate access to narrow cavities, may address these challenges and optimize vaginal surgery [15–18].

In October 2024, our team successfully performed the first robotic-assisted vaginal hysterectomy using the da Vinci® Single-Port system (RSP-vNOTES) in Europe. This study reports a case series of our initial experience with RSP-vNOTES hysterectomy. We describe the surgical technique step by step, illustrated by a surgical video, and report the per-operative and short-term postoperative outcomes of this series.

## Step-by-step surgical technique of RSP-vNOTES hysterectomy (video)

The first RSP-vNOTES hysterectomy of our center was on October 23, 2024. The patient was a 42-year woman with no previous surgical history. She had 2 vaginal deliveries and had a mobile uterus on examination. The pre-operative MRI found an anterior fibroid measuring 12 cm (FIGO stage 2–5). She received an injection of GnRH analogue 3.75 mg one month before the procedure to reduce pre-operative anemia, uterine volume and per-operative bleeding.

1. Patient positioning

Under general anesthesia, the patient was positioned in a gynecological lithotomy position. Vaginal preparation was performed using dermal betadine, and a Foley catheter was inserted for urinary drainage.

2. Initial vaginal dissection

To minimize bleeding, vaginal infiltration was carried out using lidocaine and epinephrine. A semicircular anterior colpotomy was performed, followed by vesicovaginal dissection to mobilize the bladder superiorly. The cervicovaginal vessels were coagulated and the anterior peritoneum was opened. The pouch of Douglas was accessed with scissors, and the uterosacral ligaments were coagulated and cut. These steps are the same as for classic vaginal hysterectomy.

A small vaginal retractor was placed, followed by the insertion of the da Vinci® SP access port (monotrocar). Pneumoperitoneum was established at 8 mmHg using the PneumoClear® system (Stryker, Michigan), and the patient was placed in a 15-degree Trendelenburg position.

3. Robotic docking and instrumentation setup:

The da Vinci® SP system was docked with the robotic arm oriented away from the patient’s head, ensuring optimal access to the surgical site. The camera was positioned up in the access port, bipolar forceps were placed on the left and monopolar scissors on the right. This configuration with only two robotic instruments allows for additional space so a laparoscopic grasper can be utilized by the surgical assistant.

4. Robotic surgical steps

The procedure began with the coagulation and section of the left uterine artery. Then, the broad ligament was opened up to the round ligament that was coagulated and sectioned. Salpingectomy was performed following the mesosalpinx, and the utero-ovarian ligament was sectioned since the patient wanted to preserve her ovaries. The same steps were repeated on the right side, which completely released the uterus. Throughout the procedure, the surgical assistant-maintained bladder retraction to optimize visualization and access. At the conclusion of the robotic dissection, hemostasis is checked and both ureters were clearly visualized to ensure their integrity. The robot was undocked.

5. Uterine extraction and morcellation

The uterus was extracted using a Museux clamp, and manual morcellation was performed with a scalpel. The total weight of the uterus was 800 g.

6. Vaginal closure

The colpotomy was sutured using a continuous absorbable suture and the urinary catheter was removed.

7. Postoperative course

The procedure was completed without intraoperative complications. Blood loss was minimal, and aspiration was not required. The patient was discharged the same day. Postoperative pain was minimal, with a Visual Analog Scale (VAS) score 4 h after surgery of 1.

## Materials and methods

We conducted a retrospective, single-center case series including all RSP-vNOTES hysterectomies performed in the Department of Gynecology and Obstetrics of Saint-Etienne University Hospital between October 2024 and April 2026. All cases performed during the study period are included. The only formal exclusion criteria was rectovaginal endometriosis. However, for the early of the series, patients were preferentially selected based on favorable anatomical conditions for vaginal surgery: multiparity, absence of prior cesarean delivery, absence of severe pelvic adhesions and estimated uterine weight < 1.5 kg. Suspected malignancy was not a strict contraindication; in such cases the RSP-vNOTES route was preferred when the abdominal approach was contraindicated or expected to be technically challenging (e.g., high risk of adhesiolysis from prior surgeries, intolerance to high pneumoperitoneum). As experience accumulated, selection criteria were progressively expanded to include nulliparous patients and cases with larger uterine volumes with no upper weight limitation. Priori cesarean delivery was similarly no longer considered a contraindication after the initial phase, although no such case was encountered during the study period.

Clinical and demographic data were collected from electronic medical records. These included age, body mass index (BMI), smoking status, surgical and obstetrical history (parity and mode of delivery), perioperative data (incision-to-docking time, console time, total operative time including any concomitant procedure, operating room occupancy time, concomitant procedures and uterine weight), and postoperative outcomes (complications with Clavien–Dindo grade, pain at 4 h postoperatively assessed using a VAS from 0 to 10, analgesic use before discharge, discharge prescription, and ambulatory care).

Descriptive statistical analysis was performed using Microsoft Excel®. Categorical variables were summarized as number and percentage. Given the small size of the study, continuous variables were reported as median, extreme values (minimal and maximal) and interquartile range (IQR).

This study received a favorable ethical opinion from the Comité d’Éthique pour la Recherche en Obstétrique et Gynécologie (CEROG), a national and independent Ethical Review Board for gynecologic and obstetric research in France (IRB number: #2024-GYN-0703), in accordance with its published operating procedures [19].

## Results

Between October 2024 and April 2026, 17 RSP-vNOTES hysterectomies were performed in our center by two experienced surgeons in vaginal and robotic surgery. Patient characteristics are summarized in Table 1. No patient had a history of cesarean delivery. Most patients had at least one prior vaginal delivery, while three patients (17.6%) were nulliparous.

**Table 1:** Patient characteristics

*n* = 17	Median (min, max) or *n* (%)
Age, years	45 (30, 80)
BMI^1^, kg/m^2^	23 (17, 30)
Parity	2 (0, 7)
Nulliparous	3 (17.6%)
Smoking status	5 (29%)
Previous abdominal surgery	7 (41.1%)
Surgical indication	
Fibroids	16 (94.1%)
Suspicion ovarian malignancy	1 (5.9%)

^1^Body mass index

**Fig. 1:**
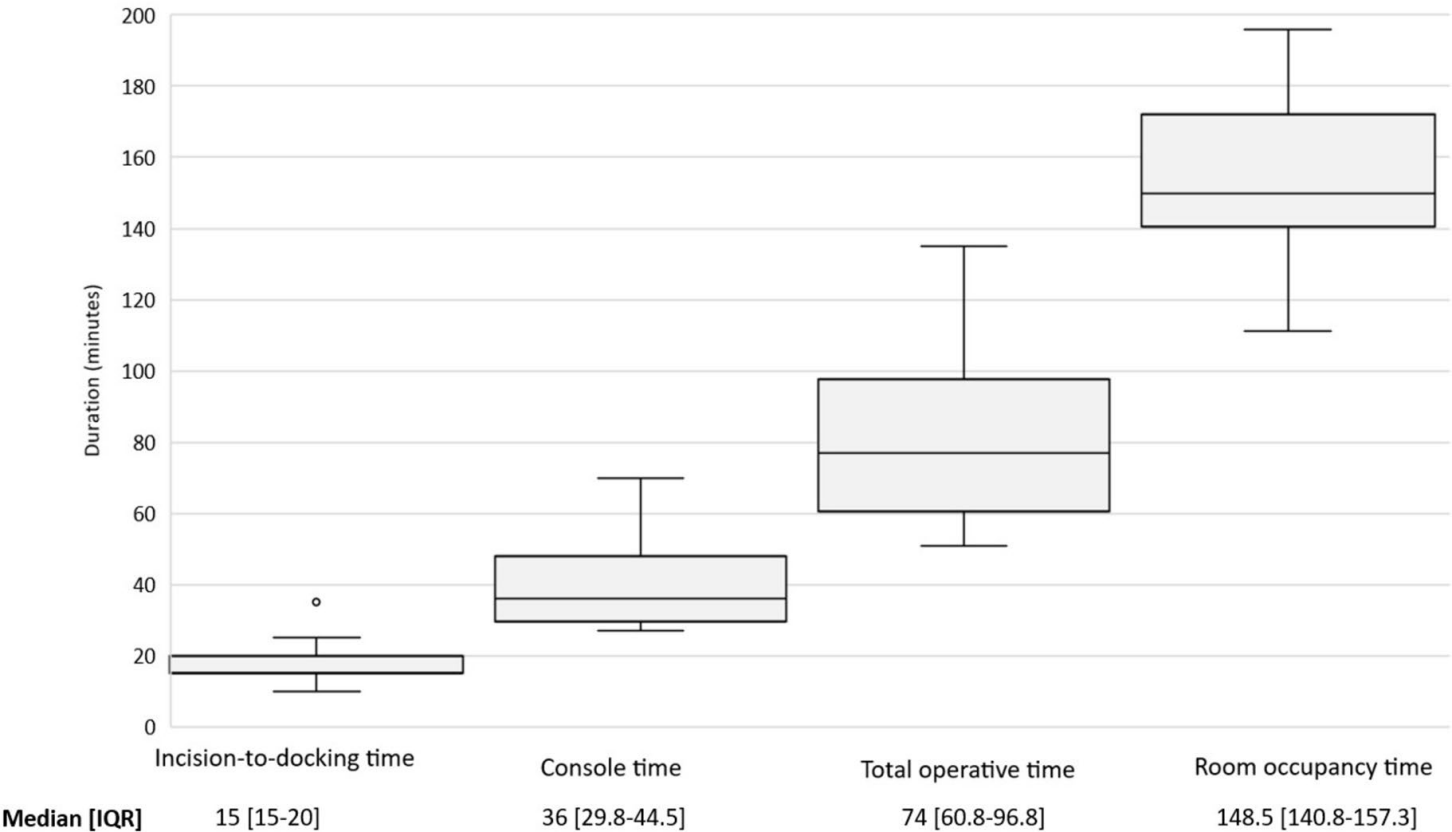
Distribution of operative time components during RSP-vNOTES hysterectomy. Box plot showing the median and interquartile range (IQR) of duration for each operative time component. Whiskers represent values within 1.5 times the IQR. Values beyond this range are displayed as individual points

The distribution of operative time components is shown in Fig. 1. Median total operative time is 74 min (60.8, 96.8) and median console time was 36 min (29.8, 44.5). The longest total operative time corresponded to a case with a suspected ovarian malignancy. In this patient, the robotic procedure was preceded by an exploratory laparoscopy using a 3-mm camera and completed by a concomitant infracolic omentectomy performed after hysterectomy through the RSP-vNOTES approach. No clear reduction in operative time over successive cases was observed. Given the small sample size and the risk of unreliable conclusion, no formal learning curve analysis was performed. For indicative purposes, the operative times of successive cases performed by the surgeon with the greatest experience in this series are presented in Table 3.

The remaining operative and postoperative outcomes are summarized in Table 2. Estimated blood loss was minimal, and no patient required a blood transfusion. Sixteen patients (94.1%) underwent same-day discharge; one patient required one night hospitalization due to anesthetic contraindication related to comorbidities. Postoperative pain was low, with a median VAS score of 0 at 4 h (0–1). Discharge prescriptions included only level 1 analgesic (paracetamol, antispasmodic) and nonsteroidal anti-inflammatory drugs (NSAIDs) for all patients. Only one patient received postoperative thromboprophylaxis for four weeks following the procedure, owing to suspected ovarian malignancy, confirmed in histopathological analysis. No postoperative complications were observed within 30 days. One patient presented to the emergency department on postoperative day 5 for vaginal bleeding; but no excessive bleeding or other complication was identified, and no intervention was required.

**Table 2:** Operative and postoperative outcomes

*n* = 17	Median [IQR^1^] or *n* (%)
Concomitant adnexectomy	2 (12.5%)
Other concomitant procedures	1 (6.3%)
Uterine weight, g^*^	370 (61, 800)
VAS^2^ pain at 4 h	0 (0–1)
VAS pain at 4 h ≥ 1	6 (29.4%)
VAS pain at 4 h ≥ 4	1 (6.3%)
Analgesic use before discharge	3 (11.8%)
Same day discharge	1 (94.1%)
Postoperative complications (30 days)	0

^*^Uterine weight was unavailable for one patient

^1^Interquartile range

^2^Visual analog scale

## Discussion

This retrospective case series reports the first European experience of robotic-assisted vaginal hysterectomy using the da Vinci® Single-Port system, with direct intravaginal insertion of the SP Access Port. All procedures were completed successfully, without conversion, intraoperative or postoperative complications. Operative and console times were acceptable, postoperative pain was minimal, and all patients were discharged on the day of surgery, supporting the feasibility and safety of this approach.

These findings are consistent with previously published studies demonstrating the feasibility of robotic-assisted vaginal hysterectomy [20–22]. Since the initial reports using multi-arm robotic platforms, robotic assistance has been shown to improve visualization, ergonomics, and instrument dexterity in confined anatomical spaces such as the vaginal canal, potentially overcoming certain limitations of conventional vaginal or laparoscopic surgery [10].

More recently, the feasibility of RSP-vNOTES hysterectomy has been reported. Guan et al. described a series of 28 RSP-vNOTES hysterectomies with acceptable perioperative outcomes, with minimal blood loss and a low complication rate, even in cases combined with endometriosis resection [23, 24]. The feasibility of RSP-vNOTES for concomitant oncological procedures, including infracolic omentectomy, has also been reported [25]. In a comparative study, RSP-vNOTES demonstrated operative time, estimated blood loss, and complication rates comparable to conventional vNOTES, with fewer conversions to abdominal approaches despite a higher rate of concomitant endometriosis procedures, suggesting that robotic assistance may facilitate the management of more complex cases [26]. However, in these studies, the SP system was introduced using an additional access device (such as a GelPOINT® or glove port) placed at the vaginal introitus. In contrast, our technique relies on direct insertion of the da Vinci® SP access port, which was specifically co-engineered with the SP robotic system to optimize instrument deployment, intracorporeal spacing, and articulation. This dedicated port offers several practical advantages: it ensures a stable and airtight seal, maintaining a consistent pneumoperitoneum with minimal smoke leakage, it provides the optimal working distance and geometry for SP instrument docking, and it simplifies the overall setup by eliminating an additional access device, potentially reducing costs and operative preparation time.

To our knowledge, this study is the first European report of RSP-vNOTES hysterectomy. Moreover, we demonstrated the feasibility of RSP-vNOTES hysterectomy with direct intravaginal insertion of the da Vinci® SP access Port without the use of supplementary access devices. This technical modification simplifies the setup and reduces docking complexity, which may contribute to relatively short operative times observed in our series, despite the presence of large uterine volumes in several cases requiring morcellation.

Patient selection appears to be a key factor for the successful implementation of robotic-assisted vaginal surgery. In the early phase of our experience, we preferentially selected patients with favorable anatomical characteristics: multiparity, no prior cesarean delivery, no severe pelvic adhesions, and estimated uterine weight < 1.5 kg. Although robotic assistance was not strictly necessary in all cases, starting with carefully selected cases was essential to safely acquire experience and standardize the technique before extending indications to more complex situations. After this initial careful selection, as experience increased, patients with prior surgical history and nulliparous patients were included, underscoring the adaptability of the approach. Importantly, robotic assistance may provide the greatest benefit in cases that fall at the boundary of conventional vaginal surgery: large or poorly mobile uteri, limited vaginal access, complex adnexal procedures, or cases where enhanced visualization and precision are required for safe dissection. In contrast, for straightforward vaginal hysterectomies in ideal candidates, conventional vaginal surgery or standard vNOTES may remain sufficient and more cost-effective.

In addition to operative efficiency, the SP robotic system offers ergonomic advantages that may be particularly relevant in vaginal surgery. Enhanced visualization, articulated instruments and stable traction may help overcome some of the limitations encountered during conventional vaginal or vNOTES approaches, especially for complex dissections or in challenging anatomical conditions. From a surgical perspective, robotic-assisted vNOTES may therefore act as a facilitating tool rather than a substitute for conventional techniques, potentially lowering the technical barrier for surgeons already experienced in vaginal surgery or laparoscopy and allowing safer performance of complex surgical steps. This may, support the extension of indications to more advanced procedures, such as endometriosis surgery, myomectomy, or even for selected oncological indications in minimally invasive surgery.

In addition, this approach may be particularly beneficial for patients with relative contraindications to laparoscopy, such as extensive intraperitoneal adhesions, or limited tolerance to prolonged steep Trendelenburg positioning). The use of low-pressure pneumoperitoneum and a limited Trendelenburg angle may reduce physiological constraints compared with standard laparoscopy, potentially facilitating access to minimally invasive surgery for a broader patient population. This aspect may be especially relevant for obese patients, who are at higher risk of complications following open surgery and may particularly benefit from less invasive alternatives.

This study has several limitations. Its retrospective design, small sample size, and single-center nature limit the generalizability of the findings. No formal learning curve analysis was performed, and no clear reduction in operative time across successive cases was observed. The learning curve for RSP-vNOTES hysterectomy has been specifically assessed in a recent series by Yang et al., which reported a plateau in operative efficiency after approximately 30 cases in surgeons experienced in both vaginal surgery and robotics [27]. However, these data may not be fully comparable to our series, as Yang et al. included predominantly cases with endometriosis and a markedly lower median uterine weight (median weight 93 g) whereas our population consisted mainly of patients with fibroid uteri and a median uterine weight of 370 g. These two clinical contexts differ substantially in terms of surgical complexity, dissection requirements and uterine extraction technique, as larger fibroid uteri frequently require morcellation. For multi-arm robotic-assisted surgery in abdominal procedures, approximately 20 cases are generally required for experienced gynecologic surgeons to achieve proficiency in docking and total operative time [28, 29]. The limited sample size of our series may explain the absence of an observable trend. Larger prospective and multicenter studies are needed to better assess learning curves, reproducibility, and long-term outcomes. Finally, the cost-effectiveness of robotic-assisted vaginal hysterectomy using the SP system remains unknown and should be evaluated in future comparative studies. However, in our series, a marked variability in operative room occupancy time was observed, which did not consistently correlate with total operative time. This suggests that optimization of workflow and operating room logistics are a necessary prerequisite before conducting robust cost-effectiveness analyses.

Despite its limitations, this study has several strengths. This first European clinical experience of RSP-vNOTES provides original and novel data in a rapidly evolving field. The study includes a standardized and reproducible surgical technique with direct intravaginal insertion of the SP Access Port, avoiding additional access devices and simplifying the setup. The detailed step-by-step description and accompanying surgical video enhance the educational value and reproducibility of the technique. Moreover, the consistency of short-term outcomes, including absence of conversion, minimal postoperative pain, same-day discharge, and lack of complications, supports the internal validity of the results. A particular strength of this series is the inclusion of cases with large uterine volumes, with a median uterine weight of 370 g and a maximum of 800 g. This goes beyond the typical range considered suitable for conventional vaginal hysterectomy and directly addresses a common criticism that vaginal or single-port approaches are only applicable to small uteri. The successful management of these larger cases demonstrates the potential versatility of RSP-vNOTES beyond strictly selected low-complexity cases.

## Conclusion

This study describes the first European cases of robotic-assisted vaginal hysterectomy using the da Vinci® Single-Port system. The successful implementation of this approach demonstrates the feasibility and safety of RSP-vNOTES even for large uterine volume, with acceptable operative times, minimal postoperative pain, and systematic same-day discharge.

These initial results suggest that the single-port robotic platform may broaden the applicability of robotic-assisted vaginal surgery by offering ergonomic advantages in confined anatomical spaces and may represent a valuable addition to minimally invasive gynecologic surgery. Larger prospective studies are required to confirm these findings, evaluate learning curves, assess cost-effectiveness, and better define the role of single-port robotic technology in minimally invasive gynecologic surgery.

## Electronic supplementary material

Below is the link to the electronic supplementary material.

Supplementary file1 (MP4 357598 KB)

## Appendix

See Table 3.

**Table 3:** Operative times of successive cases performed by the most experienced surgeon

Case number	Incision-to-docking time (minutes)	Total operative time (minutes)	Console time (minutes)	Room occupancy time (minutes)
1	15	77	35	150
2	25	60	27	180
3	20	61	29	111
4	20	135	70	196
5	35	100	46	155
6	10	71	36	147
7	15	83	39	150
8	15	55	38	120
9	20	79	35	140
10^1^	20	104	67	164
11	15	51	29	141
12	15	71	30	144
13	15	51	27	128
14	15	70	36	142
15	15	93	50	180

^1^Patient with suspected ovarian malignancy, RSP-vNOTES hysterectomy was preceded by exploratory laparoscopy and infracolic omentectomy

## References

[1] Drahonovsky J, Haakova L, Otcenasek M, Krofta L, Kucera E, Feyereisl J (2010) A prospective randomized comparison of vaginal hysterectomy, laparoscopically assisted vaginal hysterectomy, and total laparoscopic hysterectomy in women with benign uterine disease. Eur J Obstet Gynecol Reprod Biol 148:172–176 19926201 10.1016/j.ejogrb.2009.10.019

[2] McCracken G, Hunter D, Morgan D, Price J (2006) Comparison of laparoscopic-assisted vaginal hysterectomy, total abdominal hysterectomy and vaginal hysterectomy. Ulster Med J 75:54–58 16457405 PMC1891794

[3] Su H, Yen CF, Wu KY, Han CM, Lee CL (2012) Hysterectomy via transvaginal natural orifice transluminal endoscopic surgery (NOTES): feasibility of an innovative approach. Taiwan J Obstet Gynecol 51:217–221 22795097 10.1016/j.tjog.2012.04.009

[4] Kim SH, Jin CH, Hwang IT, Park JS, Shin JH, Kim DW et al (2018) Postoperative outcomes of natural orifice transluminal endoscopic surgery-assisted vaginal hysterectomy and conventional laparoscopic-assisted vaginal hysterectomy: a comparative study. Obstet Gynecol Sci 61:261–266 29564318 10.5468/ogs.2018.61.2.261 PMC5854907

[5] Lee CL, Wu KY, Tsao FY, Huang CY, Han CM, Yen CF et al (2014) Natural orifice transvaginal endoscopic surgery for endometrial cancer. Gynecol Minim Invasive Ther 3:89–92

[6] Lee CL, Huang CY, Wu KY, Hu YF, Yen CF, Han CM (2014) Natural orifice transvaginal endoscopic surgery myomectomy: an innovative approach to myomectomy. Gynecol Minim Invasive Ther 3:127–130

[7] Baekelandt J (2015) Total vaginal NOTES hysterectomy: a new approach to hysterectomy. J Minim Invasive Gynecol 22:1088–1094 26009278 10.1016/j.jmig.2015.05.015

[8] Lee CL, Liu HM, Khan S, Lee PS, Huang KG, Yen CF (2022) Vaginal natural orifice transvaginal endoscopic surgery (vNOTES) surgical staging for endometrial carcinoma: the feasibility of an innovative approach. Taiwan J Obstet Gynecol 61:345–352 35361399 10.1016/j.tjog.2022.02.026

[9] Chaccour C, Giannini A, Golia D’Augè T, Ayed A, Allahqoli L, Alkatout I et al (2023) Hysterectomy using vaginal natural orifice transluminal endoscopic surgery compared with classic laparoscopic hysterectomy: a new advantageous approach? A systematic review on surgical outcomes. Gynecol Obstet Invest 88:187–196 37231836 10.1159/000530797

[10] Lee CL, Wu KY, Su H, Han CM, Huang CY, Yen CF (2015) Robot-assisted natural orifice transluminal endoscopic surgery for hysterectomy. Taiwan J Obstet Gynecol 54:761–765 26700999 10.1016/j.tjog.2015.08.023

[11] Xu P, Zhao Z, Tian Y, Li Y, Liu Y, Ji M (2023) A retrospective analysis of robot-assisted total hysterectomy by transvaginal natural orifice transluminal endoscopic surgery. Heliyon 9:e19207 37662750 10.1016/j.heliyon.2023.e19207 PMC10474405

[12] Dapeng S, Jie C, Pu X, Jing L (2022) Transvaginal single-hole laparoscopic (V-NOTES) total hysterectomy assisted by the Da Vinci Xi system: a case report. Asian J Surg 45:1505–1506 35305873 10.1016/j.asjsur.2022.03.013

[13] Rezai S, Giovane RA, Johnson SN, Henderson CE, Liu J, Guan X (2019) Robotic natural orifice transluminal endoscopic surgery (R-NOTES) in gynecologic surgeries, a case report and review of literature. Obstet Gynecol Int J 10:287–289

[14] Thigpen B, Sun J, Guan X (2022) Robotic transvaginal NOTES: a step-by-step approach to surgical technique. Intell Surg 4:1–8

[15] Shin HJ, Yoo HK, Lee JH, Lee SR, Jeong K, Moon HS (2020) Robotic single-port surgery using the da Vinci SP® surgical system for benign gynecologic disease: a preliminary report. Taiwan J Obstet Gynecol 59:243–247 32127145 10.1016/j.tjog.2020.01.012

[16] Kwak YH, Lee H, Seon K, Lee YJ, Lee YJ, Kim SW (2022) Da Vinci SP single-port robotic surgery in gynecologic tumors: single surgeon’s initial experience with 100 cases. Yonsei Med J 63:179–186 35083904 10.3349/ymj.2022.63.2.179 PMC8819406

[17] Matsuura M, Nagao S, Kurokawa S, Tamate M, Akimoto T, Saito T (2024) Surgical outcomes of da Vinci Xi™ and da Vinci SP™ for early-stage endometrial cancer in patients undergoing hysterectomy. J Clin Med 13:2864 38792405 10.3390/jcm13102864 PMC11122509

[18] Whitmyre N, Griebel L, Buckner-Petty S, Kim KH, Yi J (2023) Outcomes of single port robotic sacrocolpopexy compared with multi-port approaches. Intell Surg 6:21–24

[19] Dabi Y, Thubert T, Fuchs F, Barjat T, Belaisch-Allart J, Ceccaldi PF, members of the CEROG committee (2022) How is functioning the Ethical Review Board « Comité d’Ethique pour la Recherche en Obstétrique et Gynécologie » (CEROG) ? J Gynecol Obstet Hum Reprod 2:102352 10.1016/j.jogoh.2022.102352 35247608

[20] Koythong T, Thigpen B, Sunkara S, Erfani H, Delgado S, Guan X (2021) Surgical outcomes of hysterectomy via robot-assisted versus traditional transvaginal natural orifice transluminal endoscopic surgery. J Minim Invasive Gynecol 28:2028–2035 34033911 10.1016/j.jmig.2021.05.014

[21] Lowenstein L, Mor O, Matanes E, Lauterbach R, Boulus S, Weiner Z et al (2021) Robotic vaginal natural orifice transluminal endoscopic hysterectomy for benign indications. J Minim Invasive Gynecol 28:1101–1106 33144242 10.1016/j.jmig.2020.10.021

[22] Yang YS (2020) Robotic natural orifice transluminal endoscopic surgery (NOTES) hysterectomy as a scarless and gasless surgery. Surg Endosc 34:492–500 31728751 10.1007/s00464-019-07115-z

[23] Guan X, Lovell D, Sendukas E (2024) Pioneering case: robotic single port (SP) transvaginal NOTES (RSP-vNOTES) for hysterectomy in ten steps. Intell Surg 7:1–6

[24] Guan X, Yang Q, Lovell DY (2024) Assessing feasibility and outcomes of robotic single port transvaginal NOTES (RSP-vNOTES) hysterectomy: a case series. J Minim Invasive Gynecol 31:1041–1049 39222841 10.1016/j.jmig.2024.08.018

[25] Zhang C, Li Q, Fang F, Guan X (2025) Using robotic single-port vNOTES for gynaecological oncology: Omentectomy in a patient with an ovarian granulosa cell tumor—a case study. J Obstet Gynaecol 45:2556279 40914934 10.1080/01443615.2025.2556279

[26] Yang Q, Lovell DY, Wu J, Zhang C, Guan X (2025) A comparative analysis of hysterectomy outcomes: robotic single-port vs. traditional transvaginal NOTES approaches. Front Med 12:1614384 10.3389/fmed.2025.1614384 PMC12615390 41244775

[27] Yang Q, Lovell DY, Guan X (2026) Surgical outcomes and learning curve of robotic single-port vaginal natural orifice transluminal endoscopic surgery (vNOTES) hysterectomy using the Da Vinci single-port system. Cureus 18(1):e102729 41777973 10.7759/cureus.102729 PMC12952751

[28] Liu J, Tan L, Thigpen B, Koythong T, Zhou X, Liu Q et al (2022) Evaluation of the learning curve and safety outcomes in robotic assisted vaginal natural orifice transluminal endoscopic hysterectomy: A case series of 84 patients. Int J Med Robot 18:e2385 35236012 10.1002/rcs.2385

[29] Torng PL, Pan SP, Hwang JS, Shih HJ, Chen CL (2017) Learning curve in concurrent application of laparoscopic and robotic-assisted hysterectomy with lymphadenectomy in endometrial cancer. Taiwan J Obstet Gynecol 56:781–787 29241920 10.1016/j.tjog.2017.10.014

